# A comparison of procedural success rate and long-term clinical outcomes between in-stent restenosis chronic total occlusion and de novo chronic total occlusion using multicenter registry data

**DOI:** 10.1007/s00392-019-01550-7

**Published:** 2019-09-24

**Authors:** Seung Hun Lee, Jae Young Cho, Je Sang Kim, Hyun Jong Lee, Jeong Hoon Yang, Jae Hyoung Park, Soon Jun Hong, Rak Kyeong Choi, Seung-Hyuk Choi, Hyeon-Cheol Gwon, Do-Sun Lim, Cheol Woong Yu

**Affiliations:** 1 Department of Cardiology, Cardiovascular Center, Mediplex Sejong Hospital, Incheon, Korea; 2 Department of Cardiovascular Medicine, Regional Cardiocerebrovascular Center, Wonkwang University Hospital, Iksan, Korea; 3 Department of Cardiology, Sejong General Hospital, Bucheon, Korea; 4 Department of Cardiology, Cardiovascular Center, Korea University Anam Hospital, Seoul, Korea; 5 Division of Cardiology, Department of Medicine, Samsung Medical Center, Sungkyunkwan University School of Medicine, Seoul, Korea

**Keywords:** Chronic coronary total occlusion, In-stent restenosis, Percutaneous coronary interventions

## Abstract

**Background:**

There have been little data about outcomes of percutaneous coronary intervention (PCI) for in-stent restenosis (ISR) chronic total occlusion (CTO) in the drug eluting stent (DES) era. This study aimed to compare the procedural success rate and long-term clinical outcomes of ISR CTO and de novo CTO.

**Methods and results:**

Patients who underwent PCI for ISR CTO (*n* = 164) versus de novo CTO (*n* = 1208) were enrolled from three centers in Korea between January 2008 and December 2014. Among a total of ISR CTO, a proportion of DES ISR CTO was 79.3% (*n* = 130). The primary outcome was major adverse cardiac events (MACEs); a composite of all-cause death, non-fatal myocardial infarction (MI), or target lesion revascularization (TLR). Following propensity score-matching (1:3), the ISR CTO group (*n* = 156) had a higher success rate (84.6% vs. 76.0%, *p* = 0.035), mainly driven by high success rate of PCI for DES ISR CTO (88.6%), but showed a higher incidence of MACEs [hazard ratio (HR): 2.06; 95% confidence interval (CI) 1.37–3.09; *p* < 0.001], mainly driven by higher prevalence of MI [HR: 9.71; 95% CI 2.06–45.81; *p* = 0.004] and TLR [HR: 3.04; 95% CI 1.59–5.81; *p* = 0.001], during 5 years of follow-up after successful revascularization, as compared to the de novo CTO group (*n* = 408).

**Conclusion:**

The procedural success rate was higher in the ISR CTO than the de novo CTO, especially in DES ISR CTO. However, irrespective of successful revascularization, the long-term clinical outcomes for the ISR CTO were significantly worse than those for the de novo CTO, in terms of MI and TLR.

**Graphic abstract:**

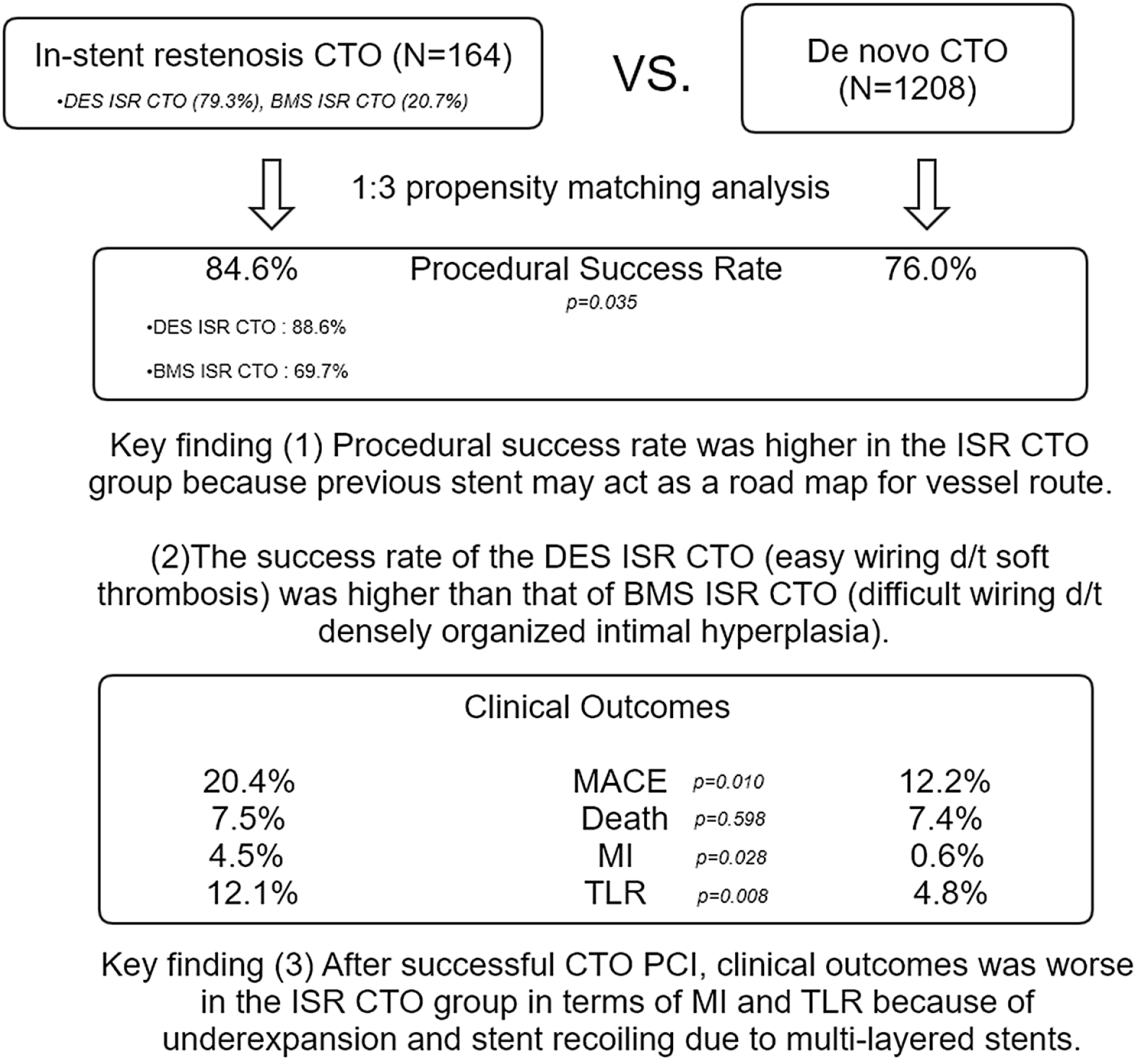

## Introduction

Revascularization of chronic total occlusion (CTO) is still one of the most challenging types of percutaneous coronary intervention (PCI) procedures. Among PCIs for CTO, PCI for in-stent restenosis (ISR) CTO is thought to be the most difficult. ISR CTO is developed as a consequence of late thrombotic stenosis and/or slow diffuse restenosis [1] and has a reported incidence of 8% of all CTOs with bare metal stents (BMSs), with the overall CTO incidence estimated to be 5–10% [1, 2]. Even though ISR CTO is rare, it causes a considerable therapeutic burden, because PCI became the standard therapy for a considerable number of coronary artery diseases; thus, the absolute quantity of ISR CTOs is currently increasing.

Several studies have reported lower PCI success rate for BMS ISR CTO as compared to de novo CTO paired with BMS [1, 3, 4]. However, there are few data regarding the procedural success rate and clinical outcomes of ISR CTO PCI in the drug-eluting stent (DES) era. Furthermore, most studies about ISR CTO in the BMS era did not have enough data to evaluate long-term clinical outcomes of ISR CTO. Therefore, in the present study, we compared the procedural success rate and long-term clinical outcomes of ISR CTO and de novo CTO when paired with DES.

## Methods

### Study population

**Fig. 1:**
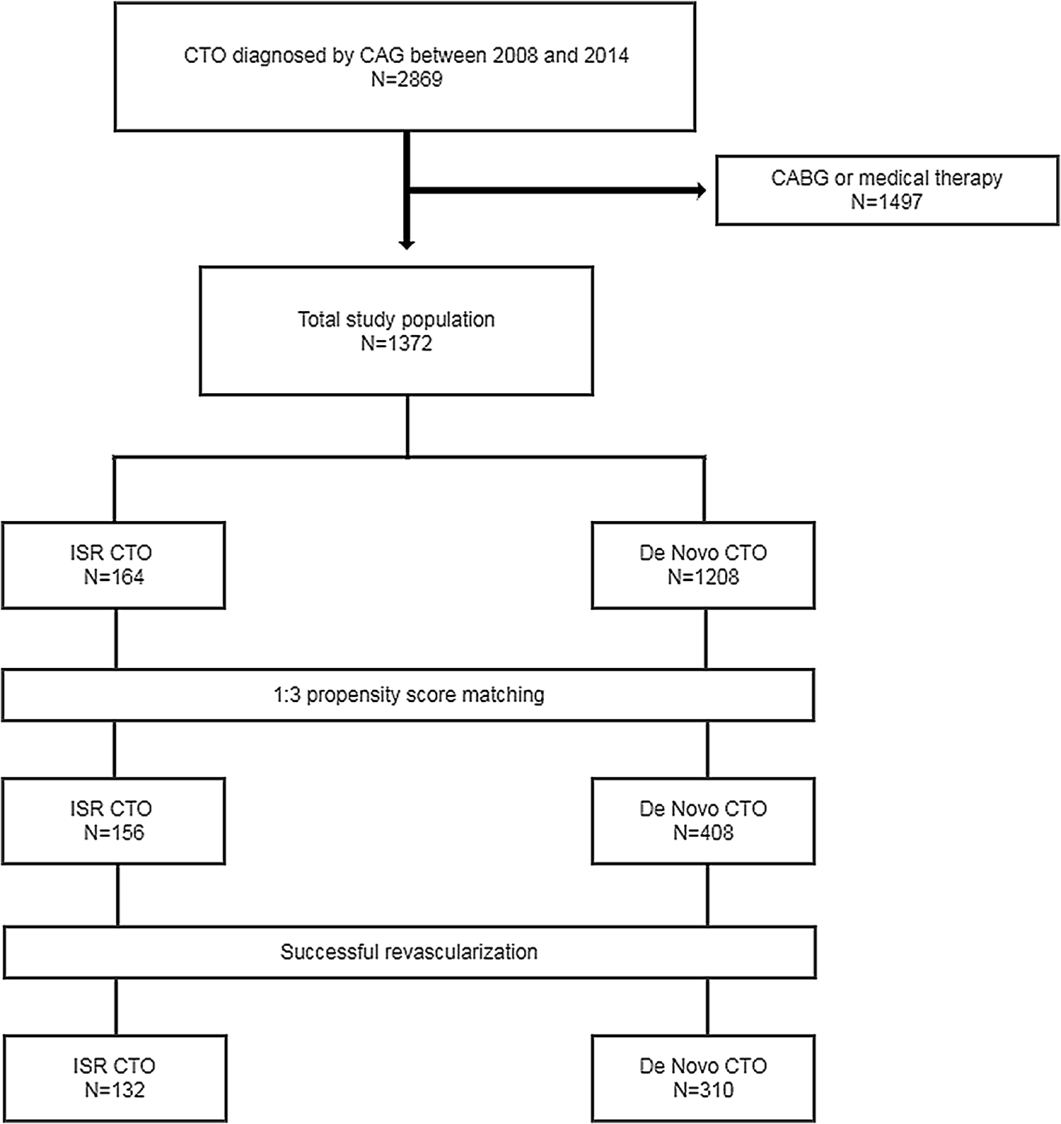
Flow chart of the study algorithm. *CABG* coronary artery bypass grafting, *CAG* coronary angiography, *CTO* chronic total occlusion, *ISR* in-stent restenosis

From January 2008 to December 2014, a total of 1372 patients underwent PCI for sole CTO at three centers in Korea. These patients were divided into the following two groups according to type of CTO: an ISR CTO group (*n* = 164, 11.9%) and a de novo CTO group (*n* = 1208, 88.1%) (Fig. 1). CTO was defined as complete occlusion of the coronary artery with thrombolysis in myocardial infarction (TIMI) anterograde flow grade of 0 and estimated occlusion duration was more than 3 months [5]. ISR CTO was defined as CTO occurring within a previously implanted stent. The occlusion duration was estimated according to myocardial infarction (MI) history for the same target vessel, previous coronary angiographic findings, and changes in electrocardiographic findings. All patients who did not take antiplatelets were pretreated with aspirin (loading dose: 300 mg) and clopidogrel (loading dose: 600 mg). The recommend antiplatelet regimen was indefinite aspirin (100 mg/day) and clopidogrel (75 mg/day) for ≥ 12 months. Clinical and procedural characteristics of both groups were retrospectively analyzed.

### Angiographic and procedural variables

We reviewed all intervention techniques and angiographic variables. An anterograde approach by coronary wire was the first option for penetrating CTO lesions. If the anterograde approach failed, we attempted a retrograde approach. Stent implantation, balloon angioplasty, and drug-eluting balloon angioplasty were performed for revascularization. Routine predilation and final post-dilation were used as standard stent implantation techniques. 79.3% of the ISR CTO group had been treated with the DES. Implanted stents during index procedures were almost all DESs (94.2%). Successful revascularization was defined as residual stenosis < 30% and TIMI flow grade ≥ 3.

### Endpoints

The primary endpoint of this study was procedural success rate, which was defined as complete revascularization of the CTO. The secondary endpoint was the incidence of major adverse cardiac events (MACEs) after 5 years of follow-ups. MACEs were defined a composite of all-cause death, non-fatal MI, or  target lesion revascularization (TLR). TLR was defined as either redo PCI or coronary artery bypass grafting because of target lesion restenosis according to objectively observable evidence of ischemia.

### Statistical analysis

All values are presented as means ± standard deviations or medians with interquartile ranges. We made comparisons between continuous variables with Student’s *t* test or the Wilcoxon rank-sum test. Categorical variables were analyzed using Fisher’s exact test or the Chi-squared test. We estimated event-free survival using the Kaplan–Meier method and compared our outcomes using the log-rank test. We also used the Cox proportional hazard model to compare the risks of adverse events between the ISR CTO and de novo CTO groups. Stepwise multiple logistic regression analysis was used to explain any associations between adverse events and other factors.

Propensity scores were calculated using logistic regression and the following baseline demographic and clinical parameters were input into our regression models as independent variables: age, sex, hypertension, diabetes mellitus, dyslipidemia, smoking, chronic renal failure on hemodialysis, history of myocardial infarction, history of coronary artery bypass grafting, history of peripheral artery disease, history of cerebrovascular accident, clinical presentation of index CTO PCI, and ejection fraction (EF). We also analyzed data with propensity score-matching including J-CTO score to reflect the complexity of CTO case. The J-CTO score was calculated for each lesion based on as previously described [6]. Propensity scores were used to perform 3:1 nearest-neighbor matching (three patients with de novo CTO to one patient with ISR CTO). After propensity score-matching, baseline variables between the two groups were compared using the paired* t* test or Wilcoxon rank test. Categorical variables were analyzed using Fisher’s exact test or the Chi-squared test. Statistical analyses were performed with SAS (version 9.2; SAS Institute Inc., Cary, NC, USA).

## Results

### Characteristics of the patients

During the study period, 1372 patients underwent PCI for CTO, with 164 of those patients undergoing PCI for ISR CTO. There were several differences in baseline clinical variables between the de novo CTO group and the ISR CTO group (Table 1). As compared to the de novo CTO group, the ISR CTO group was younger (59.1 years ± 10.3 years vs. 62.2 years ± 10.9 years, *p* < 0.001). One hundred and fourteen of the ISR CTO patients were male, which was different from the de novo CTO group (69.5% vs. 77.6%, *p* = 0.027). The prevalences of hypertension, diabetes mellitus, and current smoking habit were higher in the de novo CTO group. Patients in the ISR CTO group had a more frequent history of previous MI as compared to patients in the de novo CTO group (42.7% vs. 16.2%, *p* < 0.001). When receiving treatment of index CTO PCI, the proportions of patients who were clinically diagnosed with stable angina, acute coronary syndrome (ACS), or other were not statistically different between the two groups (*p* = 0.530). The proportion of patients with left ventricular (LV) dysfunction, which was defined as an EF < 40%, were not significantly different between the two groups (12.2% of ISR CTO group vs. 10.9% of de novo CTO group, *p* = 0.724).

**Table 1:** Baseline characteristics of ISR and de novo groups of patients with CTO

	Total population			Propensity-matched population		
	ISR CTO (*n* = 164)	De novo CTO (*n* = 1208)	*p* value	ISR CTO (*n* = 156)	De novo CTO (*n* = 408)	*p* value
Age (years)	59.1 ± 10.3	62.2 ± 10.9	< 0.001	59.5 ± 10.3	60.2 ± 11.0	0.594
Male gender (%)	114 (69.5)	938 (77.6)	0.027	111 (71.2)	304 (74.5)	0.483
Diabetes mellitus (%)	50 (30.5)	500 (41.4)	0.010	48 (30.8)	142 (34.8)	0.420
Hypertension (% )	88 (53.7)	759 (62.8)	0.029	87 (55.8)	237 (58.1)	0.687
Current smoking (%)	36 (22.0)	381 (31.5)	0.016	36 (23.1)	101 (24.8)	0.760
Dyslipidemia (%)	81 (49.4)	594 (49.3)	1.000	77 (49.4)	206 (50.5)	0.884
Previous MI (%)	70 (42.7)	196 (16.2)	< 0.001	64 (41.0)	138 (33.8)	0.134
Previous PCI (%)	78 (100)	284 (20.9)	< 0.001	–	–	–
Previous CVA (%)	9 (5.5)	89 (7.4)	0.474	9 (5.8)	30 (7.8)	0.633
Previous CABG (%)	7 (4.3)	11 (0.9)	0.001	4 (2.6)	7 (1.7)	0.756
Previous PAD (%)	6 (3.7)	30 (2.5)	0.524	5 (3.2)	14 (3.4)	1.000
CRF on HD (%)	11 (6.7)	72 (6.0)	0.840	10 (6.4)	31 (7.6)	0.761
eGFR (mL/min/1.73 m²)	68.3 ± 33.2	68.4 ± 37.2	0.983	68.1 ± 38.0	70.5 ± 34.1	0.746
LDL (mg/dL)	68.9 ± 48.7	92.8 ± 53.2	0.008	68.9 ± 48.7	89.4 ± 52.2	0.045
Medication at last follow up						
Aspirin (%)	154 (93.9)	1139 (94.3)	0.983	146 (93.6)	389 (95.3)	0.528
Dual antiplatelet (%)	138 (84.1)	1057 (87.5)	0.245	132 (84.6)	353 (86.5)	0.823
Statin (%)	136 (82.9)	923 (76.4)	0.077	130 (83.3)	316 (77.5%)	0.155
Clinical presentation			0.530			0.416
Stable angina (%)	78 (47.5)	695 (57.5)		76 (48.7)	231 (56.6)	
ACS (%)	67 (40.9)	383 (31.7)		61 (39.1)	132 (32.3)	
Other (%)	19 (11.6)	130 (10.8)		19 (12.2)	45 (11.1)	
LVEF, %	55.3 ± 13.6	57.4 ± 12.7	0.064	55.6 ± 13.7	57.1 ± 12.6	0.341
LV dysfunction (EF < 40%) (%)	20 (12.2)	132 (10.9)	0.724	18 (11.5)	45 (11.0)	0.982

Data are presented as *n* (%) or mean ± standard deviation

*ACS* acute coronary syndrome, *CABG* coronary artery bypass graft, *CTO* chronic total occlusion, *CVA* cerebrovascular accident, *MI* myocardial infarction, *PCI* percutaneous coronary intervention, *LAD* left anterior descending artery, *LVEF* left ventricular ejection fraction

Angiographic variables are shown in Table 2. The most common target CTO lesion site was the LAD (43.3%) and then the RCA (38.4%) in the ISR CTO group. CTOs were located in the LAD and the RCA with similar distribution in the de novo CTO group. Prior to propensity score-matching, the mean J-CTO score and the proportion of the J-CTO score ≥ 3 were higher in the ISR CTO group; however, this characteristic became comparable between the two groups after propensity score-matching.

**Table 2:** Angiographic and procedural characteristics of the study subjects

	Total population			Propensity-matched population		
	ISR CTO (*n* = 164)	De novo CTO (*n* = 1208)	*p* value	ISR CTO (*n* = 156)	De novo CTO (*n* = 408)	*p* value
CTO lesion site						
LAD	71 (43.3)	463 (38.3)	0.151	69 (44.2)	164 (40.1)	0.249
LCX	30 (18.3)	252 (20.9)	0.011	27 (17.3)	85 (20.8)	0.104
RCA	63 (38.4)	493 (40.8)	0.180	60 (38.5)	159 (39.1)	0.916
CTO lesion length (mm)	16.4 ± 11.1	19.6 ± 14.1	0.586	15.6 ± 11.1	18.5 ± 13.2	0.713
Syntax score	17.8 ± 11.1	17.3 ± 10.5	0.654	18.1 ± 11.2	16.7 ± 10.2	0.299
J-CTO score			< 0.0001			0.107
0	12 (7.3%)	221 (18.3%)		11 (7.0%)	60 (14.7%)	
1	47 (28.6%)	396 (32.8%)		46 (29.4%)	117 (28.7%)	
2	49 (29.9%)	276 (22.9%)		45 (28.9%)	105 (25.7%)	
≥ 3	56 (34.2%)	315 (26.0%)		54 (34.7%)	126 (30.9%)	
Mean J-CTO score	2.07 ± 1.21	1.68 ± 1.27	< 0.001	2.08 ± 1.22	1.89 ± 1.30	0.097
Number of diseased vessels	1.65 ± 0.85	2.04 ± 0.88	< 0.001	1.63 ± 0.80	2.00 ± 0.89	0.001
Proximal to mid CTO	110 (67.0)	883 (73.1)	0.127	104 (66.7)	311 (76.2)	0.287
Collateral flow grade (%)			< 0.001			< 0.001
0	30 (18.2)	39 (3.2)		29 (18.5)	13 (3.1)	
1	35 (21.3)	267 (22.1)		33 (21.1)	92 (22.5)	
2	67 (40.8)	541 (44.7)		65 (41.6)	176 (43.1)	
3	32 (19.5)	361 (29.8)		29 (18.5)	127 (31.1)	
Type of intervention, *n* (%)			0.165			0.458
Drug-eluting stent	146 (89.1)	1127 (93.3)		142 (91.1)	378 (92.7)	
Balloon	18 (10.9)	81 (6.7)		14 (8.9)	30 (7.3)	
Total stent length	30.2 ± 14.9	30.2 ± 17.1	0.982	29.4 ± 13.7	31.1 ± 18.0	0.441
Total number of stent used	1.80 ± 0.5	1.84 ± 0.6	0.194	1.74 ± 0.5	1.85 ± 0.72	0.167
Maximum stent diameter	2.94 ± 0.4	3.08 ± 0.9	0.021	2.95 ± 0.4	3.16 ± 1.4	0.061
Fluoroscopic time (min)	40.1 ± 64.4	25.5 ± 26.1	0.313	–	–	–
Contrast volume (ml)	190.3 ± 89.2	216.2 ± 123.6	0.332	–	–	–

Data are presented as *n* (%) or mean ± standard deviation

*CABG* coronary artery bypass graft, *CTO* chronic total occlusion, *CVA* cerebrovascular accident, *MI* myocardial infarction, *PCI* percutaneous coronary intervention, *LAD* left anterior descending artery, *LVEF* left ventricular ejection fraction

Fluoroscopic time during index PCI (40.1 min ± 64.4 min in ISR CTO vs. 25.5 min ± 26.1 min in de novo CTO, *p* = 0.313) and the amount of contrast agent used during index PCI (190.3 ml ± 89.2 ml in ISR CTO vs. 216.2 ml ± 123.6 ml in de novo CTO, *p* = 0.332) were not different between the two groups.

After propensity score-matching, the overall study population included 564 patients whose clinical variables were matched with those of the controls (Table 1). There were no significant differences between the ISR CTO group and the de novo CTO group concerning baseline clinical characteristics and mean J-CTO score.

### Clinical outcomes

Successful revascularization was achieved in 84.2% of the ISR CTO group and 78.4% of the de novo CTO group, respectively, which were not significantly different from one another (*p* = 0.110). During a 5-year period of follow-up, 45 MACEs occurred in the ISR CTO group and 144 MACEs occurred in the de novo CTO group [27.4% vs. 11.9%; hazard ratio (HR): 2.17; 95% CI 1.55–3.04; *p* < 0.001; Table 3]. The all-cause death rate was not different between the two groups. However, the prevalences of MI (5.4% vs. 1.4%; HR: 3.57; 95% CI 1.59–8.02; *p* = 0.002) and TLR (14.0% vs. 4.3%; HR: 2.91; 95% CI 1.77–4.79; *p* < 0.001) were higher in the ISR CTO group than in the de novo CTO group.

**Table 3:** Clinical outcomes of the study subjects after successful PCI in a total population and propensity-matched population during 5 years

	Total population			Propensity-matched population			Subjects who underwent successful revascularization after propensity score-matching		
	ISR CTO (*n* = 164)	De novo CTO (*n* = 1208)	*p* value	ISR CTO (*n* = 156)	De novo CTO (*n* = 408)	*p* value	ISR CTO (*n* = 132)	De novo CTO (*n* = 310)	*p* value
MACE (%)	45 (27.4)	144 (11.9)	< 0.001	35 (22.4)	51 (12.5)	< 0.001	27 (20.4)	38 (12.2)	0.010
Death (%)	18 (10.9)	87 (7.2)	0.211	13 (8.3)	29 (7.1)	0.266	10 (7.5)	23 (7.4)	0.598
MI (%)	9 (5.4)	17 (1.4)	0.002	8 (5.1)	8 (1.9)	0.077	6 (4.5)	2 (0.6)	0.028
TLR (%)	23 (14.0)	53 (4.3)	< 0.001	19 (12.1)	16 (3.9)	0.001	16 (12.1)	14 (4.5)	0.008

Data are presented as *n* (%) or mean ± standard deviation

*ISR* in-stent restenosis, *CTO* chronic total occlusion, *MI* myocardial infarction, *PCI* percutaneous coronary intervention, *TLR* target lesion revascularization

**Fig. 2:**
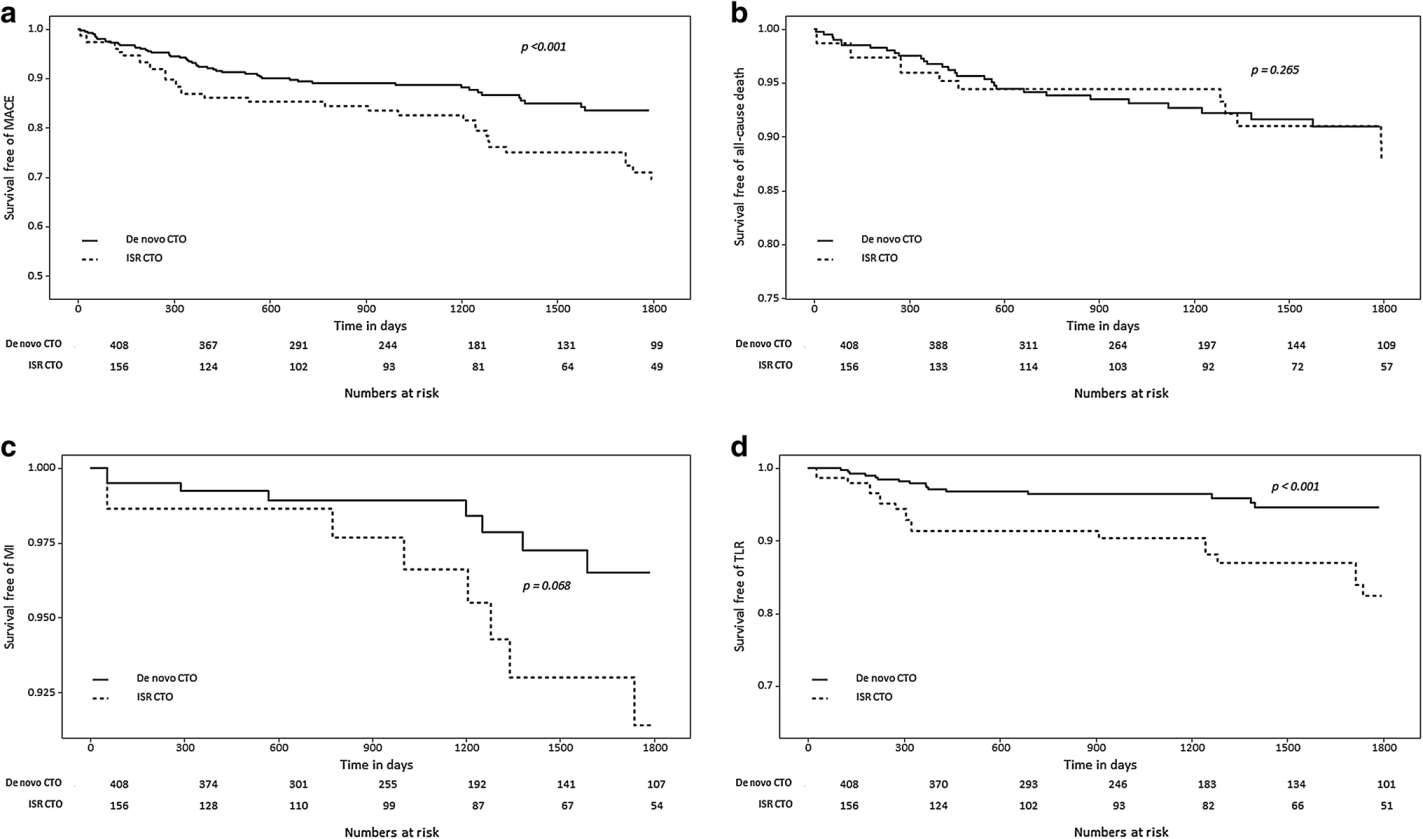
Kaplan–Meier curves for outcomes according to type of CTO after propensity score-matching. Curves for **a** MACEs as a composite of all-cause death, MI, and TLR. **b** All-cause death. **c** MI. **d** TLR

After matching subjects according to propensity score, we found that the ISR CTO group had a higher success rate (84.6% in the ISR CTO group vs. 76.0% in the de novo CTO group, *p* = 0.035). The procedural success rate based on the type of initially implanted stent was significantly higher in DES ISR CTO than in BMS ISR CTO (88.6% vs. 69.7%, *p* = 0.016). The ISR CTO group was also associated with a higher incidence of MACEs (22.4% in the ISR CTO group vs. 12.5% in the de novo CTO group; HR: 2.06; 95% CI 1.37–3.09; *p* < 0.001; Fig. 2), which resulted from a significant increase in the MI and TLR rates.

**Fig. 3:**
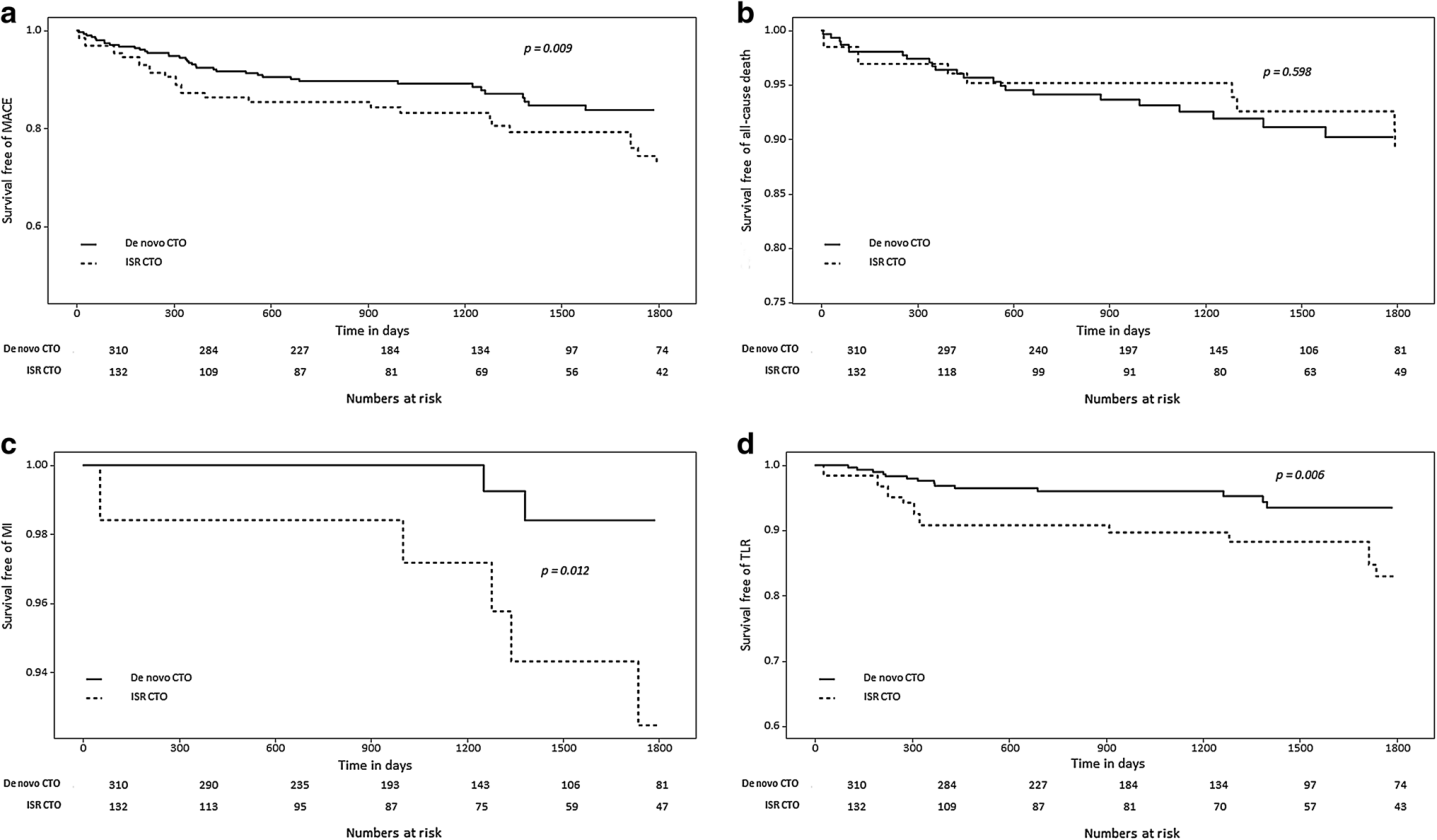
Kaplan–Meier curves for outcomes according to type of CTO that underwent successful PCI after propensity score-matching. Curves for **a** MACEs as a composite of all-cause death, MI, and TLR. **b** All-cause death. **c** MI. **d** TLR

MACEs after successful revascularization in propensity-matched populations occurred in 27 patients (20.4%) in the ISR CTO group and in 38 patients (12.2%) in the de novo CTO group (*p* = 0.010; Fig. 3), a finding that was mainly driven by MI (4.5% in the ISR CTO group vs. 0.6% in the de novo CTO group, *p* = 0.028) and TLR (12.1% in the ISR CTO group vs. 4.8% in the de novo CTO group, *p* = 0.008).

Table 4 shows the results of multiple stepwise logistic regression analysis assessing the relationship between MACEs and several potential risk factors. In study subjects who experienced successful revascularization, LV dysfunction (adjusted HR: 2.84; 95% CI 1.77–4.55; *p* < 0.001), CRF on hemodialysis (adjusted HR: 2.79; 95% CI 1.62–4.82; *p* < 0.001), and ISR CTO (adjusted HR: 2.06; 95% CI 1.37–3.09; *p* < 0.001) were independent predictors of 5-year MACEs.

**Table 4:** Independent predictors of MACEs in the subjects (*n* = 1085) after successful revascularization by multiple stepwise logistic regression analysis

	Adjusted HR (95% CI)	*p* value
LV dysfunction	2.84 (1.77–4.55)	< 0.001
CRF on hemodialysis	2.79 (1.62–4.82)	< 0.001
ISR CTO	2.06 (1.37–3.09)	< 0.001

This analysis included age, gender, diabetes mellitus, hypertension, ISR CTO, ACS, current smoking, CRF on hemodialysis, dyslipidemia, Hx. of CABG, Hx. of MI, HX. of CVA, Hx. of PAD, family Hx. of CAD, and LV dysfunction. For abbreviations, see Tables 1, 2, and 3

## Discussion

The present study has two major findings. First, procedural success rate was higher in the ISR CTO group than in the de novo CTO group in the propensity-matching analysis. Second, irrespective of successful revascularization of CTO, the long-term clinical prognosis of the ISR CTO group was significantly worse than that of the de novo CTO group, especially in terms of TLR.

ISR CTO lesions is still a challenging subset for successful CTO revascularization. In earlier period, the reported success rate of PCI for ISR CTO was lower than that of PCI for de novo CTO. Abbas reported a revascularization success rate for BMS ISR CTO of 63% (49 of 78 patients) and for de novo CTOs of 75% (164 of 235 patients) from 2002 to 2003 [3]. Werner et al. reported a success rate of 70% for DES ISR CTO and one of 85% for de novo CTO between 2005 and 2007 [1]. Along with the advancement of technique and devices for CTO PCI in the later period, the procedural success rate for DES ISR CTO between 2008 and 2010 was improved up to 86% in a separate study by Christopoulos et al. [7]. Herein, success rate of PCI in our study was high in DES ISR CTO and low in BMS ISR CTO, which are similar to the result of previous studies, and overall success rate of PCI for ISR CTO was higher than those of de novo CTO in propensity score-matching analysis. Mean J-CTO score, fluoroscopic time, and contrast volume was not different significantly between the two groups after propensity-matching, which demonstrate that the difficulty level of the procedure was similar between the groups.

Contrary to a previous BMS ISR CTO investigation [3], our study estimated a superior success rate for ISR CTO as compared to de novo CTO, which could be attributed to our larger number of DES ISR CTO cases. The mechanism for ISR development is different between DES and BMS [8, 9]. BMS ISR develops over a long period with smooth-muscle, cell-rich homogeneous tissue [10, 11]. Inside the stent, intimal hyperplasia is densely organized without fissuring, leading to ISR. This results in difficult guidewire penetration and balloon dilatation. In contrary to BMS ISR, DES ISR is made of hypo-cellular and proteoglycan-rich tissue [12, 13]. Thrombosis, the main mechanism of DES ISR, produces relatively softer ISR lesions than does BMS ISR. The current study showed that DES ISR CTO accounted for 79.3% of total ISR CTO, which might partially explain why success rate of PCI for ISR CTO was higher than that of de novo CTO. Therefore, the selection strategy for optimal wire passage of CTO segments should be different between BMS ISR CTO and DES ISR CTO.

The duration of ISR formation was also different between the two stent types [14]. Intimal hyperplasia formation in BMS usually peaks at 6 months and continues slowly afterward, while thrombosis in DES cases occurs within a relatively short period [15]. DES ISR can occur suddenly up to several years after implantation [12]. It is not clear how the formation time of CTO influences the difficulty of the procedure; however, when abundant collateral vessels are formed over a longer ISR period in BMS cases, the target vessel route can be confirmed and a retrograde procedure may be simpler to perform in these instances [16, 17].

As compared to PCI for de novo CTO, ISR CTO has both technical advantages and disadvantages. While wiring during PCI for de novo CTO frequently fails because of difficulty in finding the precise vessel route, it can be easier during PCI for ISR CTO, because the previous stent acts as a road map of the target vessel [18]. Furthermore, a previously implanted stent prevents vessel dissection or injury during PCI for ISR CTO.

Although vessel routes can be easily found in ISR CTO cases, the wire frequently gets caught by the strut of the initial stent or is undermined by the previous stent. Subintimal tracking and wire reentry to the true lumen are also not easily performed in ISR CTO cases, and it can be hard to advance a new stent in cases in which a deformity in the previous stent developed during balloon passage. Balloon and stent under expansion can also occur in ISR CTO cases. In addition, a newly implanted stent is often in conflict with a previous stent [4, 19].

Clinical outcomes of ISR CTO were worse than those of de novo CTO after successful revascularization with respect to MACEs, which was mainly driven by higher incidence of TLR and tended to be associated with higher incidence of MI, in our data. ISR has been shown not to be a benign clinical condition [8, 20, 21]. Rathore et al. [8] reported that approximately 18% of patients in their study with ISR presented with ACS, with 2% presenting with MI. Additionally, MI incidence of BMS and first-generation DES was about 10% in the study by Magalhaes et al. [21]. Our results showed that the TLR rate of ISR CTO group at 5 years of follow-up was higher than in the de novo CTO group, a finding which was deemed to be associated with a higher incidence of MI of the ISR CTO group.

We also found that stenting for ISR CTO was an important risk factor of MI and TLR. Very long or multiple stent implantations for full lesion coverage are known to be risk factors of restenosis after PCI [22, 23]. Therefore, the most likely explanation for our findings is that multi-layered stenting is associated with abnormal vessel reaction and thrombus formation. In addition, stent recoiling generated from two stent layers might increase the risk of underexpansion. As there are concerns about worse clinical outcomes due to multi-layered stent as we found, so interventionist might consider using of drug-coated balloons rather than DES in the treatment of ISR CTO lesions [24, 25]. Severe impairment of vasomotor tone occurs after PCI for CTO [26], which results in changes in blood-flow dynamics and subsequent high susceptibility to thrombus formation and/or atherosclerotic progression. Therefore, precise analysis of tissue characteristics such as calcium distribution and anatomical morphology of CTO segments such as lumen size, vessel size, lesion length, plaque burden, and optimal lesion preparation is extremely important for successful wiring and optimal stenting, which might be relevant to long-term stent patency. Modern imaging techniques, including coronary computed tomography angiogram, intravascular ultrasound, and optical coherence tomography, enable us to conduct examinations prior to and during PCI for ISR CTO [27, 28]. Due to the guidance using these advanced imaging technologies, results of wire passage and optimal stent implantation are improving [29–31].

### Study limitations

Regarding limitations of our study, first, the nonrandomized nature of the registry data could have resulted in selection bias. Several baseline characteristics were significantly different between the two groups, and the decision to perform PCI for CTO was made by the physician. Although we performed a propensity score-matched analysis to adjust for these potential confounding factors, we were not able to correct for unmeasured variables. Second, adverse clinical events were not centrally adjudicated in our registries. All events were identified by the patients’ physicians and were confirmed by the principal investigator at each hospital. Third, because of the retrospective nature of our registry, we could not thoroughly identify all instances of changes to patients’ medical therapy strategies during follow-up. Fourth, our study population had a high prevalence of multi-vessel disease, so our results might not be generalizable to populations with less severe disease. Fifth, given the observed clinical event rates, a properly powered study would require a larger sample population. Accordingly, this study was considerably underpowered, and our subgroup analysis was not conclusive. Finally, even though past and present evidence suggest that the most important predictor of successful PCI for CTO is surgeon experience and skill, we were unable to evaluate this.

## Conclusion

The procedural success rate was higher in the ISR CTO than in the de novo CTO, especially in DES ISR CTO. However, irrespective of successful revascularization, the long-term clinical prognosis of the ISR CTO was significantly worse than that of the de novo CTO, in terms of MI and TLR.
